# Association of Bone Loss with the Upregulation of Survival-Related Genes and Concomitant Downregulation of Mammalian Target of Rapamycin and Osteoblast Differentiation-Related Genes in the Peripheral Blood of Late Postmenopausal Osteoporotic Women

**DOI:** 10.1155/2015/802694

**Published:** 2015-02-10

**Authors:** Elena V. Tchetina, Karina A. Maslova, Mikhail Y. Krylov, Valery A. Myakotkin

**Affiliations:** ^1^Genetics Department, Nasonova Research Institute of Rheumatology, Moscow 115522, Russia; ^2^Clinical Immunology Department, Nasonova Research Institute of Rheumatology, Kashirskoye Shosse 34A, Moscow 115522, Russia; ^3^Janssen Pharmaceutical Companies of Johnson and Johnson, Toronto, ON, Canada N1K 1A5

## Abstract

We aimed to identify bone related markers in the peripheral blood of osteoporotic (OP) patients that pointed toward molecular mechanisms underlying late postmenopausal bone loss. Whole blood from 22 late postmenopausal OP patients and 26 healthy subjects was examined. Bone mineral density (BMD) was measured by DXA. Protein levels of p70-S6K, p21, MMP-9, TGF*β*1, and caspase-3 were quantified by ELISA. Gene expression was measured using real-time RT-PCR. OP registered by low BMD indices in late postmenopausal patients was associated with a significant upregulation of autophagy protein *ULK1*, cyclin-dependent kinase inhibitor *p21*, and metalloproteinase *MMP-9* gene expression in the blood compared to the healthy controls and in a significant downregulation of *mTOR* (mammalian target of rapamycin), *RUNX2*, and *ALPL* gene expression, while expression of *cathepsin K, caspase-3, transforming growth factor* (*TGF*)* β1, interleukin-* (*IL-*) *1β*, and *tumor necrosis factor α* (*TNFα*) was not significantly affected. We also observed a positive correlation between *TGFβ1* and *RUNX2* expression and BMD at femoral sites in these patients. Therefore, bone loss in late postmenopausal OP patients is associated with a significant upregulation of survival-related genes (*ULK1* and *p21*) and *MMP-9*, as well as the downregulation of *mTOR* and osteoblast differentiation-related genes (*RUNX2* and *ALPL*) in the peripheral blood compared to the healthy controls.

## 1. Introduction

Osteoporosis (OP) is characterized by a low bone mass, poor bone quality, and an increased propensity to fracture. Osteoporotic fractures, which are often associated with decreased bone mineral density (BMD), are a major public health problem in the aging population [1]. However, the molecular mechanisms underlying OP related bone destruction are not completely understood at the present time. Recently it has been suggested that these mechanisms involve altered osteoblast and osteoclast differentiation and function [2].

Excessive bone degradation in OP is thought to result from an increased activity of resorptive osteoclasts, which express proteases such as metalloproteinase (MMP-9) and cathepsin K. Cathepsin K, a papain-like cysteine protease, is considered a key player in the process of bone resorption [3, 4]. Osteoclast differentiation and function are regulated by the tumor necrosis factor *α* (TNF*α*), receptor activator of nuclear factor kappa B ligand (RANKL), and macrophage colony-stimulating factor (M-CSF). These three cytokines may promote osteoclast survival by signaling through mammalian target of rapamycin (mTOR) as a common target [5]. mTOR is recognized as an evolutionarily conserved central coordinator of fundamental biological processes involving cell cycle progression, translational control, ribosomal biogenesis, transcription control, and autophagy [6]. Indeed, recent animal studies have shown that mTOR is capable of regulating osteoclastogenesis, while the downregulation of mTOR reduced osteoclast production and activity [7, 8].

Differentiation-related phenotypic alterations in osteoblasts include changes in specific gene expression and corresponding protein synthesis during the course of proliferation, extracellular matrix maturation, and mineralization [9, 10]. Initially, actively proliferating cells express cell cycle and cell growth regulating genes such as Runt-related transcription factor (RUNX) 2, an osteoblast transcriptional regulator, which also controls the expression of major bone matrix protein genes [11]. Osteoblast maturation is associated with production of the bone matrix from regularly and densely packed collagen fibrils and high mineralization. At this stage, the mechanical properties and composition of the bone matrix are regulated by transforming growth factor (TGF)*β*1 [12]. A decline in proliferative activity is accompanied by the enhanced expression of the CDK inhibitor p21(CIP1/WAF1) (p21) and alkaline phosphatase [9, 13]. As a caspase-3 substrate, physiological amounts of p21 are responsible for the apoptotic activity observed in many cell lines, including osteoblasts [14].

Alterations in mTOR signaling may also be involved in osteoblast phenotypic conversions [15, 16]. It has been shown that upregulation of mTOR signaling was associated with an increase in BMP2, RUNX2, and TGF*β* expression in preosteoblasts [17]. In contrast, mTOR downregulation by rapamycin inhibited osteoblast proliferation and differentiation and reduced RUNX2, sialoprotein, and osterix gene expression, alkaline phosphatase activity, and mineralization capacity [18].

Furthermore, the downregulation of mTOR is accompanied by increased autophagy in many cell types [19]. Autophagy is a physiological cellular mechanism that degrades and recycles proteins to maintain an adequate amino acid level for survival purposes. It involves the formation of cytosolic double membrane vesicles (autophagosomes) associated with the upregulation of ULK (hATG)1–15 gene expression. Hyperautophagic conditions are capable of promoting caspase-dependent apoptotic cell death [20]. Recently, the importance of autophagy in OP development and progression was described [21, 22]. In particular, the regulation of autophagy (ROA) pathway was shown to be significantly associated with wrist and arm BMD [23]. Moreover, autophagic proteins are important for the generation of the osteoclast ruffled border, their secretory function, and bone resorption [24].

The interplay of anabolic and catabolic factors, which are involved in major metabolic pathways and are associated with osteoporotic bone loss, is currently unclear. Clinical studies might help clarify this issue. However, bone specimens from postmenopausal OP patients are largely unavailable. On the other hand, it is well established that the number of genes simultaneously expressed in various cell types is higher in developmentally related tissues. As immune and bone cells originate from the mesoderm and the formation of bone and adaptive immune systems are phylogenetically closely related, these systems might involve identical regulatory cytokines and growth factors [25]. Moreover, it was suggested recently that peripheral blood mononuclear cells (PBMCs) contain substantial numbers of T-lymphocytes, which are capable of producing the proinflammatory cytokines IL-1 and TNF*α*, which could be involved in postmenopausal bone loss [26, 27].

Here, we compared expression of the genes associated with bone cell differentiation and bone resorption (*RUNX2, TGFβ*1, ALPL, TNF*α*, IL-1*β*, *MMP-9, *and* cathepsin K*) and the genes responsible for global cell survival and functioning (*mTOR, p21*,* caspase-3*, and* ULK1*) in the peripheral blood of late postmenopausal OP patients and healthy subjects. We observed a significant upregulation of* ULK1, p21*, and* MMP-9* gene expression in the blood of the OP women compared to the healthy controls. This was associated with a significant downregulation of* mTOR*,* RUNX2,* and alkaline phosphatase (*ALPL*) gene expression, while the expression of* cathepsin K, caspase-3, IL-1β*, and* TNFα* was not significantly different than that of the healthy subjects. We also observed a positive correlation between* TGFβ1* and* RUNX2* expression and the BMD at femoral sites in these patients. We concluded that late postmenopausal osteoporosis is associated with an upregulation of cell survival as well as a downregulation of cell growth/proliferation and osteoblast differentiation/function related gene expression as registered in the peripheral blood cells.

## 2. Patients and Methods

### 2.1. Ethics

The study protocol was approved by the Local Committee on the Ethics of Human Research and informed consent was obtained from all subjects.

### 2.2. Patients

The study included 22 consecutive, unrelated, late postmenopausal, Russian women with idiopathic osteoporosis who visited the outpatient clinic of the Nasonova Research Institute of Rheumatology. The average age of the OP patients was 66.1 ± 7.2 years, with a range of 53–76 years of age. The average menopause duration was 18.0 ± 4.8 years, with a range of 10–30 years. Individuals with disorders known to cause abnormalities in bone metabolism, including diabetes mellitus, renal diseases, rheumatoid arthritis, and thyroid, parathyroid, and other endocrinological diseases, were excluded from the study. Women that had taken drugs, such as estrogen, progesterone, glucocorticoids, bisphosphonates, and alfacalcidol, were also excluded.

Twenty-six age-matched postmenopausal healthy volunteers (average age 63.0 ± 12.2 years, with a range of 49–78 years of age) who did not have any serious diseases, including osteoarthritis, and had not taken drugs known to affect bone and calcium metabolism were also recruited in the Moscow area. The study protocol was approved by the Local Committee on the Ethics of Human Research and informed consent was obtained from all subjects. The study was conducted in full accordance with the current version (2008) of the Declaration of Helsinki.

### 2.3. Measurement of BMD

BMD of the lumbar spine (L1–L4), femoral neck, femoral trochanter, femoral intertrochanter, Ward triangle, and total femur was measured by dual-energy X-ray absorptiometry (DXA) using a QDR-4500w instrument (Hologic, USA) at the Nasonova Institute of Rheumatology. The diagnosis of osteoporosis was based on the criteria recommended by the World Health Organization [28], which included a *T*-score < −2.5 SD. According to this test, all the OP subjects examined in this study were diagnosed with osteoporosis (Table 1).

### 2.4. Peripheral Blood Mononuclear Cell (PBMC) Isolation

Peripheral blood (10 mL) was collected in Vacutainer tubes containing ethylenediaminetetraacetic acid (EDTA) (BDH, England). The blood samples were taken in a standardized manner in the morning (between 07:00 AM and 09:00 AM). Whole blood fractionation was performed using a Ficoll density gradient. Upon centrifugation the peripheral blood mononuclear cells (PBMCs) located in the interphase were collected and washed twice in phosphate-buffered saline (PBS) [29]. The obtained cell fractions were frozen and kept at −70°C prior to protein extraction.

### 2.5. Quantification of p70-S6K, p21, MMP-9, TGF*β*1, and Caspase-3 Protein Levels

The concentrations of total p70-S6K (KHO0571), p21WAF1/Cip1 (KHO5421), MMP-9 (KHС3061), active caspase-3 (KHO1091) (Invitrogen, Camarillo, CA, USA), and TGF*β*1 (BMS249/4) (eBioscience, Vienna, Austria) were determined in the isolated PBMCs using commercially available enzyme linked immunosorbent assay (ELISA) kits according to the manufacturer's instructions. For mTOR protein expression, we evaluated the levels of p70-S6K, an mTOR direct target for phosphorylation, which is used as a readout of mTOR activity [30, 31], as mTOR ELISA kits are not available in Russia.

The results were expressed per *µ*g of protein measured in the PBMC lysates. The PBMC lysates were obtained using Cell Extraction Buffer containing 10 mM Tris, pH 7.4, 100 mM NaCl, 1 mM EDTA, 1 mM EGTA, 1 mM NaF, 20 mM Na_4_P_2_O_7_, 20 mM Na_3_VO_4_, 1% Triton X-100, 10% glycerol, 0.1% SDS, and 0.5% deoxycholate (Invitrogen, Camarillo, CA, USA) supplemented with Protease Inhibitor Cocktail (Sigma-Aldrich, Inc., St. Louis, USA) and 1 mM PMSF (Sigma-Aldrich, Inc., St. Louis, USA) according to the manufacturer's instructions. The total protein concentration in the cell lysates was quantified using the Bradford method [32].

### 2.6. Total RNA Isolation and Reverse Transcriptase (RT) Reaction

For detection of gene expression total RNA was isolated from 100 *µ*L of whole blood immediately after withdrawal using Ribo-zol-A kit (InterLabService, Moscow, Russia) in accordance with the manufacturer's recommendations. Total RNA had an A_260/290_ > 1.9. The RT reaction was performed using a Reverta kit containing M-MLV Reverse Transcriptase, random hexanucleotide primers, and total RNA according to the manufacturer's recommendations (InterLabService, Moscow, Russia).

### 2.7. Real-Time Quantitative PCR

The following premade primers and probes were used for the TaqMan assay (Applied Biosystems, Foster City, CA, USA):* mTOR* (Hs00234522_m1),* Unc-51-like kinase 1* (*ULK1*) (Hs00177504_m1),* p21WAF1/Cip1* (*p21*) (Hs00355782_m1),* caspase-3* (Hs00263337_m1),* TNFα* (Hs00174128_m1),* TGFβ1 *(Hs99999918_m1)*, RUNX2 *(Hs00231692_m1)*, ALPL *(Hs00758162_m1),* cathepsin K* (Hs00166156_m1),* MMP-9 *(Hs00234579_m1), and* IL-1β* (Hs00174097_m1).  *β-actin* was used as an endogenous control.

The quantification of gene expression was conducted using a 7300 Real-Time PCR System (Applied Biosystems, Foster City, CA, USA) as described previously [33]. Briefly, 1 *μ*L of RT product was subjected to real-time PCR in a 15 *μ*L total reaction mixture containing 7.5 *μ*L of TaqMan Universal PCR Master Mix (Applied Biosystems), 900 nM sense and antisense primers, 50 nM probe, and template cDNA. After a single step of 50°C for 2 min and an initial activation at 95°C for 10 min, the reaction mixtures were subjected to 40 amplification cycles (15 s at 95°C for denaturation and 1 min of annealing and extension at 60°C).

Relative mRNA expression was determined using the delta-delta C_T_ method, as detailed by the manufacturer guidelines (Applied Biosystems) [34]. The delta C_T_ value was calculated by subtracting the C_T_ value for the housekeeping *β-actin* gene from the C_T_ value for each sample. A delta-delta C_T_ value was then calculated by subtracting the delta C_T_ value of the control (each healthy patient) from the delta C_T_ value of each OP patient. Each PCR was performed in duplicate. Three “no template” controls were consistently negative for each reaction.

### 2.8. Statistical Analysis

A Kolmogorov-Smirnov normality test showed that the data were distributed according to a Gaussian distribution curve. Therefore, for statistical evaluations, Pearson's rank correlations and unpaired Student's *t*-test were used for comparisons between the control subjects and the OP patients. As we compared only a control sample with OP sample in case of each gene and did not perform any multiple testing, no correction for multiple testing was made. Quantitative data were expressed as the mean ± SD. The Statistica 6 Software (StatSoft, Tulsa, OK, USA) was used for all statistical analyses.* P *values less than 0.05 were considered significant.

## 3. Results

### 3.1. Whole Blood Gene Expression

**Figure 1:**
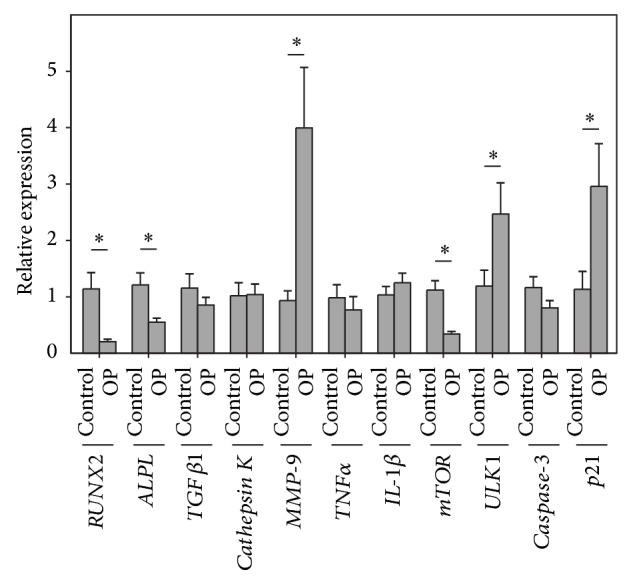
Relative expression* of RUNX2, ALPL, TGF*β*1, cathepsin K, MMP-9, TNF*α*, IL-1*β*, mTOR, ULK1, caspase-3, *and* p21* genes with reference to *β-actin* gene in the peripheral blood of 22 postmenopausal OP patients compared to 26 healthy age-matched control women (control). Each point represents the mean ± SD. Significant differences as compared to the control are indicated by asterisks.

Examination of the gene expression in the whole blood of OP patients revealed that* mTOR, RUNX2, *and* ALPL* genes were significantly downregulated compared to the healthy subjects (Figure 1). In contrast the expression of the* p21*, autophagy-related* ULK1, *and* MMP-9* genes was significantly upregulated in the same OP patients. No significant changes were observed in the expression of* caspase-3*,* TNF*α*, IL-1*β*, TGF*β*1*, and* cathepsin K*.

### 3.2. Protein Levels of p70-S6K, p21, MMP-9, TGF*β*1, and Caspase-3 in Isolated PBMC

**Figure 2:**
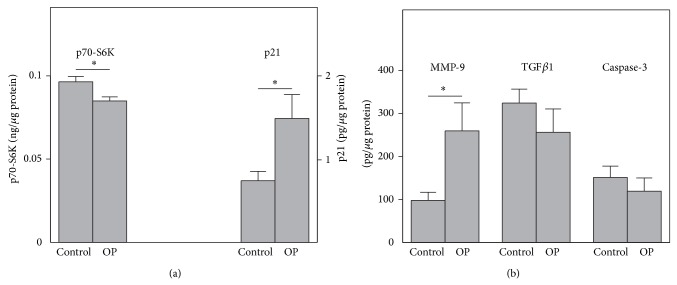
Protein concentrations of p70-S6K, p21 (a), MMP-9, TGF*β*1, and caspase-3 (b) measured by ELISA in the PBMCs from OP patients (*n* = 22) compared with the control subjects (*n* = 26). Asterisks indicate significant differences from the healthy control subjects.

To further investigate the clinical significance of* mTOR, p21*,* MMP-9, TGF*β*1, *and* caspase-3* gene expression in the whole blood of OP patients, we analyzed the protein levels of total p70-S6K serine/threonine kinase (a direct target of mTOR phosphorylation [30, 31]), p21, MMP-9, TGF*β*1, and active caspase-3 in the PBMC fraction. These studies showed that OP patients demonstrated significantly lower p70-S6K protein concentrations in PBMCs compared to the healthy subjects (Figure 2). At the same time, p21 and MMP-9 protein levels were significantly increased, while no changes were observed in the amounts of active caspase-3 and TGF*β*1 in PBMCs of the OP patients, as compared to the healthy patients.

### 3.3. Association of Gene Expression with BMD

The analysis of bivariate correlations using Pearson's correlation coefficient for the expression of the examined genes showed that* mTOR, TGF*β*1, RUNX2, TNF*α*, IL1*β*, cathepsin K, caspase-3, *and* p21* positively (*P* < 0.05) correlated with each other (Table 2). However, no correlation was observed between* ULK *and* ALPL* gene expression and the other genes.

The expression of some of the examined genes also significantly correlated with BMD in the OP patients (Table 2). A positive correlation was noted between* TGF*β*1* and* RUNX2* gene expression with BMD of the total femur and its compartments (trochanter and Ward triangle) in the examined OP patients.

## 4. Discussion

Recently, the skeletal and immune systems have been thought to interact more than previously believed, and this interaction primarily involves regulatory aspects [35]. Moreover, T- and B-lymphocytes are thought to be key regulators of osteoclast and osteoblast formation, lifespan, and activity [26]. Therefore, we suggested that changes in gene and protein expression in the peripheral blood cells of osteoporotic patients might also be associated with the disease related metabolic alterations observed in the bone cells. Here, we used the PBMCs of late postmenopausal OP patients and healthy controls to investigate the differences in the expression of genes related to the regulation of bone cell differentiation and bone resorption.

We show that the expression of osteoclast differentiation and function related genes was either unaltered (*cathepsin K, TNFα,* and* IL-1β*) or upregulated (*MMP-9*) in the peripheral blood of the examined OP women compared to the healthy subjects. Previous studies have also noted that osteoporotic women had serum IL-1*β* levels similar to those of normal controls [36]. In addition, no difference in the frequency of* TNFα* expression was observed in the bone tissue of OP versus healthy women [37]. Upregulation of* MMP-9* gene and protein expression in the PBMCs of the examined OP women is supported by the in situ hybridization results of others, which showed an increase in* MMP-9* mRNA in osteoporotic bone tissue versus the normal controls [3]. However, some studies also reported decreased* MMP-9* expression in the bone of the postmenopausal OP patients [38]. Although* MMP-9* expression was upregulated in the PBMCs of the examined OP women, its proteolytic activity in osteoclasts might be limited by the unaltered expression of* cathepsin K,* as both proteinases are required to be equally expressed for tissue proteolysis [39]. At the same time, the osteoclast proteolytic activity might not be directly pursued in the peripheral blood as we did not observe any correlation of the examined osteoclast differentiation and function related genes with the BMD indices.

In contrast, significant downregulation of osteoblast differentiation-related* RUNX2* and* ALPL* gene expression in the PBMCs of OP women compared to the healthy age-matched controls, as well as a positive association of* RUNX2 *gene expression with the BMD at femoral sites, might indicate a significant reduction in bone formation during late postmenopausal OP. Previously, a decrease in* RUNX2* and* ALPL* expression was also observed in the bone tissue of OP patients compared to healthy subjects [38, 40]. In addition, a lower* RUNX2 *expression and a subsequently increased bone loss were observed after treating of rodents with bone resorptive agents, such as rosiglitazone [41]. Therefore, bone loss during late postmenopausal OP might be associated with decreased bone formation rather than with increased bone resorption activity.

Our study also shows that late postmenopausal bone loss is associated with alterations in the non-tissue-specific gene and protein expression in the peripheral blood. It is well established that many human diseases occur when the precise regulation of cell growth (cell mass/size) and proliferation (rates of cell division) is compromised [42]. Therefore, the significant downregulation of* mTOR* gene expression that was associated with the decreased expression of osteoblast differentiation and function related genes (*RUNX2* and* ALPL*) in the PBMCs of late postmenopausal OP women compared to healthy subjects was not surprising. Our observation is supported by the previous observations that the upregulation of mTOR signaling is associated with an increase in bone forming activity in rats and cultured human osteoblast-like cells [43, 44]. In contrast, systemic administration of the mTOR inhibitor FK506 caused dramatic OP in animals [45, 46], which was accompanied by the downregulation of* RUNX2* expression [47]. Moreover, immunosuppressant FK506 therapy resulted in severe bone loss and an increase in fracture incidence in 65% of the patients [48]. Furthermore, a positive correlation between* mTOR* gene expression and the genes responsible for osteoblast (*RUNX2* and* TGFβ1*) and osteoclast (*cathepsin K, TNFα*, and* IL-1β*) related differentiation and activity indicates the association of bone loss during OP with a general declination of cell growth and proliferation activity in the examined women.

Significant upregulation of autophagy-related* ULK1* gene expression in the examined OP women might involve an increase in cell maintenance, as autophagy has been shown to be associated with increased survival in many cell types [49]. Indeed, increased osteoclast survival was previously observed in association with rapid bone loss in glucocorticoid-induced OP in animal studies [50]. Other animal studies have shown that the downregulation of autophagy resulted in the inhibition of MCPIP (zinc finger CCCH-type containing 12A) induced expression of osteoclastic markers in monocytic osteoclast precursors [51]. Therefore, increased osteoclast survival in the examined OP patients may explain the excessive bone loss in the absence of the upregulation of the genes related to osteoclast differentiation and activity, such as* cathepsin K* and* TNFα*.

The upregulation of* p21,* in association with the* mTOR* inhibition, which was observed in the PBMCs of the examined OP women, was also noted in cultured cells from various lineages following treatment with the mTOR inhibitor, rapamycin [52]. On the other hand, the upregulation of mTOR signaling in response to oscillatory shear stress was associated with a decreased expression of* p21* in human osteoblast-like cells [44]. In addition, our observation of decreased* RUNX2* gene expression associated with* p21* upregulation in the blood of OP patients is supported by a previous study that showed* p21* promoter repression by RUNX2 in osteoblast lineage cells [53]. Several other studies have also demonstrated an association between p21 upregulation and the inhibition of osteoblast proliferation and differentiation and an increase in the mononuclear precursor cell differentiation into osteoclasts [54–56].

## 5. Conclusion

Here, we show that peripheral blood cells of postmenopausal OP women exhibit significant upregulation of the* ULK1*,* p21*, and* MMP-9* genes. This is associated with a significant downregulation of* mTOR, RUNX2*, and* ALPL* gene expression. Although the peripheral levels of cytokines and other factors regulating bone turnover do not always reflect the levels observed within the bone, the correlation of* TGF*β*1* and* RUNX2* gene expression with BMD suggests that the expression of these gene counterparts in the bone tissue might be similarly affected. Therefore, bone resorption in late postmenopausal OP might be associated with decreased bone formation and an increased survival of the bone degrading cells. Further experiments should be performed to confirm our observations.

## Figures and Tables

**Table 1:** Average BMD values (±SD) in the examined postmenopausal osteoporotic women and healthy subjects.

	Healthy controls	*T*-score	OP patients	*T*-score	*P*
	(*n* = 26)		(*n* = 22)		
Lumbar spine L1–L4	1.052 ± 0.06	0.064 ± 0.62	0.712 ± 0.06	−3.07 ± 0.60	<0.001
Femoral neck	0.857 ± 0.06	0.064 ± 0.59	0.584 ± 0.08	−2.407 ± 0.71	<0.001
Trochanter	0.765 ± 0.11	0.633 ± 1.08	0.531 ± 0.07	−1.703 ± 0.74	<0.001
Intertrochanter	1.163 ± 0.10	0.525 ± 0.70	0.830 ± 0.16	−1.696 ± 1.05	<0.001
Ward triangle	0.727 ± 0.10	−0.042 ± 0.91	0.378 ± 0.07	−2.839 ± 1.24	<0.001
Total femur	1.156 ± 0.12	0.533 ± 0.70	0.700 ± 0.11	−1.853 ± 1.11	<0.001

**Table 2:** Correlation coefficients and their significance (*P*) are shown for the expression of cell growth and differentiation-related genes in relation to BMD in postmenopausal osteoporotic women.

	mTOR	TGF*β*1	RUNX2	TNF*α*	IL-1*β*	Caspase-3
RUNX2	**0.768**	**0.768**				
	(*P* < 0.001)	(*P* < 0.001)				

TNF*α*	**0.664**		**0.504**			
	(*P* = 0.005)		(*P* = 0.03)			

IL-1*β*	**0.424**	**0.696**	**0.496**	**0.494**		
	(*P* = 0.06)	(*P* < 0.001)	(*P* = 0.01)	(*P* = 0.03)		

Caspase-3	**0.666**	**0.481**	**0.519**	**0.867**	**0.637**	
	(*P* = 0.003)	(*P* = 0.03)	(*P* = 0.01)	(*P* < 0.001)	(*P* = 0.002)	

p21	**0.580**	**0.868**	**0.680**	**0.715**	**0.675**	**0.715**
	(*P* = 0.009)	(*P* < 0.001)	(*P* = 0.001)	(*P* = 0.002)	(*P* = 0.001)	(*P* = 0.001)

TGF*β*1	**0.632**					
	*P* = 0.004					

Cathepsin K	**0.542**			**0.516**		
	(*P* = 0.03)			(*P* = 0.07)		

Lumbar spine						

Femoral neck						

Trochanter			**0.548**			
			(*P* = 0.01)			

Ward triangle			**0.516**			
			(*P* = 0.02)			

Total femur		**0.524**	**0.605**			
		(*P* = 0.02)	(*P* = 0.005)			
